# The nucleolar size is associated to the methylation status of ribosomal DNA in breast carcinomas

**DOI:** 10.1186/1471-2407-14-361

**Published:** 2014-05-22

**Authors:** Maria Giulia Bacalini, Annalisa Pacilli, Cristina Giuliani, Marianna Penzo, Davide Treré, Chiara Pirazzini, Stefano Salvioli, Claudio Franceschi, Lorenzo Montanaro, Paolo Garagnani

**Affiliations:** 1 Department of Experimental, Diagnostic and Specialty Medicine, University of Bologna, Bologna, Italy; 2 Personal Genomics S.r.l., Verona, Italy; 3 Interdepartmental Center “L. Galvani”, University of Bologna, Bologna, Italy; 4 Centro Interdipartimentale di Ricerche sul Cancro ‘Giorgio Prodi’-CIRC, University of Bologna, Bologna, Italy; 5 Department of Biological, Geological and Environmental Sciences, University of Bologna, Bologna, Italy; 6 Applied Biomedical Research Center, S. Orsola-Malpighi Polyclinic, Bologna, Italy

## Abstract

**Background:**

There is a body of evidence that shows a link between tumorigenesis and ribosome biogenesis. The precursor of mature 18S, 28S and 5.8S ribosomal RNAs is transcribed from the ribosomal DNA gene (rDNA), which exists as 300–400 copies in the human diploid genome. Approximately one half of these copies are epigenetically silenced, but the exact role of epigenetic regulation on ribosome biogenesis is not completely understood. In this study we analyzed the methylation profiles of the rDNA promoter and of the 5’ regions of 18S and 28S in breast cancer.

**Methods:**

We analyzed rDNA methylation in 68 breast cancer tissues of which the normal counterpart was partially available (45/68 samples) using the MassARRAY EpiTYPER assay, a sensitive and quantitative method with single base resolution.

**Results:**

We found that rDNA locus tended to be hypermethylated in tumor compared to matched normal breast tissues and that the DNA methylation level of several CpG units within the rDNA locus was associated to nuclear grade and to nucleolar size of tumor tissues. In addition we identified a subgroup of samples in which large nucleoli were associated with very limited or absent rDNA hypermethylation in tumor respect to matched normal tissue.

**Conclusions:**

In conclusion, we suggest that rDNA is an important target of epigenetic regulation in breast tumors and that rDNA methylation level is associated to nucleolar size.

## Background

Epigenetic regulation of ribosomal DNA (rDNA) locus has a pivotal role in orchestrating ribosome biogenesis. Human cells contain about 400 copies of the ribosomal RNA (rRNA) genes organized as tandem, head-to-tail repeats [1,2], which are located in the fibrillar centers and the dense fibrillar component of the nucleolus [3]. Each unit is ~43 kb long and includes the 47S rRNA encoding sequence (~13 kb) and a non-transcribed intergenic spacer (~30 kb). In physiological conditions, around half of these copies is allelically inactivated through a combination of epigenetic mechanisms including late replication time [4], specific repression factors [5,6] and methylation of rDNA promoter. rDNA promoter includes a core promoter region, extending from −50 to +20 in respect to the transcription starting site (TSS), and an upstream control element (UCE) at −200 in respect to TSS. In humans, but not in rodents, both the UCE and the core promoter are CpG rich regions, classifiable as CpG islands, which usually show a complex methylation pattern [7,8] that can affect rRNA expression [9,10].

Bisulfite sequencing of clonal rDNA promoters has been used to characterize rDNA methylation status in several pathological conditions. Hypermethylation of rDNA promoter was described in brain from Alzheimer’s disease [11] and suicide subjects [12], while methylation levels of 18S and 28S 5’ regions were decreased in white blood cells from systemic lupus erythematosus subjects [13]. rDNA hypermethylation occurs during aging [14], and accordingly accelerated methylation of ribosomal regions was shown in fibroblasts from subjects affected by Werner syndrome [15]. The analysis of rDNA methylation in tumor samples appears to be in this context of extreme interest. Ribosome biogenesis is a limiting factor in sustaining the increased demand for protein synthesis, a prerequisite for cell growth and cell proliferation [16,17], and, as consequence, the rate of ribosome production is notably enhanced in cancer cells. rDNA promoter was found hypomethylated in respect to corresponding normal tissue in human hepatocellular carcinomas [7] but not in prostate cancer [18]. On the contrary, Yan and colleagues used methylation-sensitive Southern blotting to show increased rDNA methylation in patients with breast cancer compared to the normal control tissue; rDNA hypermethylation resulted also in association with specific tumor features such as the negativity of oestrogen receptors and poor tumor differentiation status [19].

In this study we analyzed methylation levels of three different regions within rDNA genes (the promoter and 5’ regions of 18S and 28S sequences). In order to precisely define rDNA methylation profiles in breast cancer we used the MassARRAY EpiTYPER assay, a more sensitive and quantitative method compared to Southern blot and to clonal sequencing. Furthermore, we investigated a possible correlation between the methylation status of single CpG sites, ribosomal biogenesis and the available clinical and bio-pathological parameters in order to define its possible impact in the biological and clinical behavior of the tumors.

## Methods

### Patient materials, characterization and total DNA extraction

The study was approved by the St Orsola-Malpighi Hospital’s ethical review board (approval number 75/2011/U/TESS). All volunteers provided written, informed consent. Sixty eight breast carcinomas were selected from a series of consecutive patients who had undergone surgical resection for primary breast carcinoma at the Surgical Department of the University of Bologna between 2005 and 2012, on the sole basis of frozen tissue availability. For forty five patients we collected both tumoral and non tumoral adjacent tissues (later on named normal tissue). Each patient’s clinical information was recorded and correspondent tissue was histologically characterized by a team of clinical pathologists to define its bio-pathological features according to standard criteria for both clinical parameters and TNM (Tumour-Nodal-Metastasis) classification [20]. The expression of the oncosuppressor protein p53, Estrogen and Progesteron Receptors (ER and PR respectively) and proliferative markers ki67 was measured by experts after specific immunohistochemical (IHC) staining at the Operative Unit of Anatomy, Pathological Histology of the Sant’Orsola-Malpighi University Hospital in Bologna using NovoLinkTM Polymer Detection System (Novocastra Laboratories Ltd.) and following the manufacturer’s instruction. For IHC analysis, the following mouse monoclonal primary antibodies were used: p53 (1:400, Novocastra); ER (1:450, DakoCytomation, Glostrup, Denmark); PR (1:400, Novocastra); ki67 (1:200, Novocastra). Silver staining of Nucleolar Organizer regions (AgNORs) was performed as described below. Specimen collection and tissue analyses were approved by the Bologna University Ethical Committee on human tissues research. Tissues were preserved at −80°C until use. A piece of 60 mg for each sample was minced in liquid nitrogen and then lysed for total DNA extraction using buffers provided with NucleoSpinTissue Columns kit (Macherey Nagel) and following the manufacturer’s instructions.

### EpiTYPER assay for quantitative DNA methylation analysis

Quantitative DNA methylation analysis of rDNA locus was performed using the EpiTYPER assay (Sequenom). Briefly, 1000 ng of DNA were bisulphite converted using the EZ-96 DNAMethylation Kit (Zymo Research Corporation) as previously described [21]. 10 ng of bisulphite-treated DNA were PCR-amplified using the following primers: *Ribo* forward: AGGAAGAGAGGTGTGTTTTGGGGTTGATTAGAG; *Ribo* reverse: CAGTAATACGACTCACTATAGGGAGAAGGCTAAAACCCAACCTCTCCAAC; *18S* forward: AGGAAGAGAGGTTTGTTGTTTTTTTTGGATGTGG; *18S* reverse: CAGTAATACGACTCACTATAGGGAGAAGGCTCCTTACCTACCTAATTAATCCTACCAA; *28S* forward: AGGAAGAGAGGGTATTTAGTTTTAGATGGAGTTTATTATT; *28S* reverse: CAGTAATACGACTCACTATAGGGAGAAGGCTAAAAAAAACTAACCAAAATTCCC. For each gene, CpG sites with missing values in more than 20% of the samples were removed, as well as samples with missing values in more than 20% of CpG sites.

### Selective nucleolar staining

Five-micron sections were processed to perform the silver staining to visualize the nucleolar organizer regions and the argyrophilic proteins according to the guidelines of the “International committee on AgNOR quantitation” [22]. Tissues were deparaffinized in xylene and rehydrated in decreasing concentrations of ethanol and distilled water. After antigen retrieval in citrate buffer pH 6.0 at 120°C, 1 atm for 21 minutes, the sections were then incubated in silver nitrate solution in a dark for 13 min at 37°C. The silver staining solution consisted of one part of silver nitrate (Diapath) and two parts of 2% gelatin (Sigma) in 1% formic acid (Carlo Erba) solution. Ultra pure distilled water was used for preparation of all solutions. The sections were then washed in distilled water, dehydrated in graded alcohol and xylene and cover slipped. The tissue was then ready for counts. After silver-staining, the NORs can be easily identified as black dots exclusively localized throughout the nucleolar area. Silver stained section was examined through a light microscope using Image-Pro Plus6 software (Media Cybernetics). The morphometric analysis was performed on a cell by cell basis of at least 200 nuclei and the mean nucleolar area was calculated.

The best cutoff value for the nucleolar size variable was obtained by the receiver operating characteristic curve and corresponded to the value of 5 μm^2^.

### Statistical analysis

DNA methylation values resulting from EpiTYPER assay are reported as continuous values ranging from 0 (0% of methylation) to 1 (100% of methylation). All analyses were performed in R 2.14. For continuous parameters, the following thresholds were used in ANOVA and chi-squared tests: age > 50 years; diameter > 20 mm; p53 > 10% of positivity; Ki67 > 20% of positivity; nucleolar size > 5 μm^2^. p-values < 0.05 were regarded as statistically significant.

## Results

### Characterization of rDNA target regions

To profile the rDNA methylation status in breast cancer, genomic DNA was extracted from 68 breast carcinomas samples; for 45 of them, pair-matched normal tissues were available.

**Figure 1:**
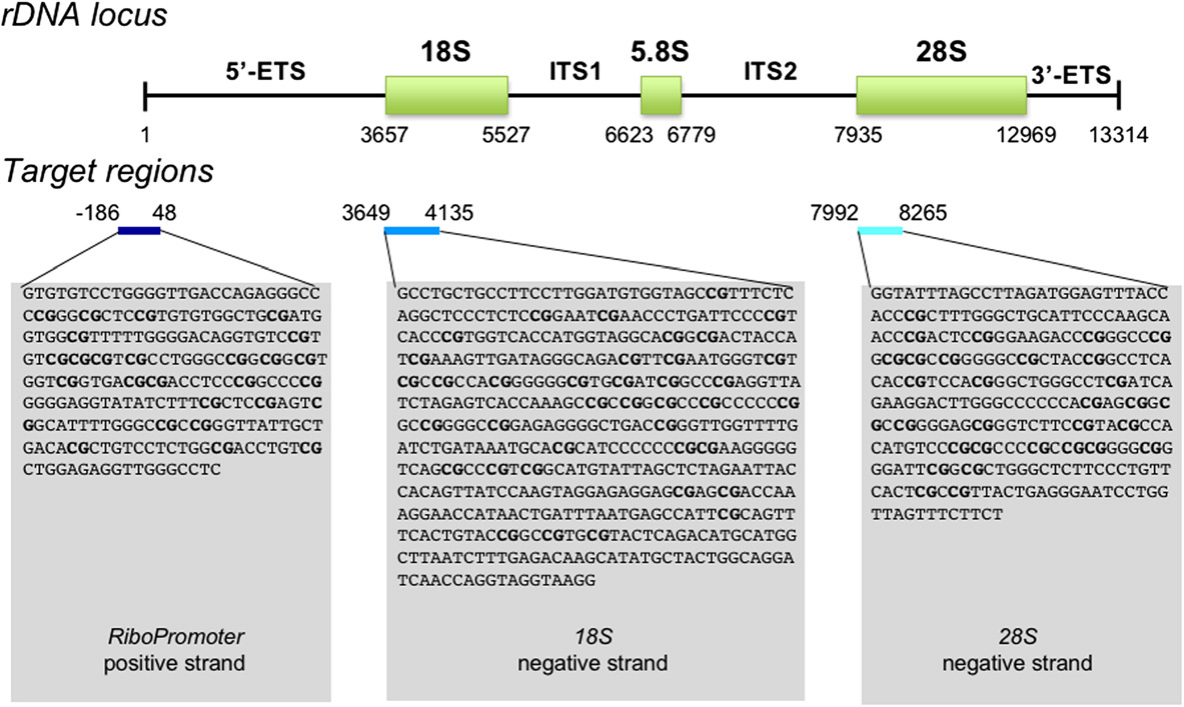
**Location of the target regions selected for DNA methylation analysis within the rDNA locus.** The picture reports a schematic representation of the rDNA locus and the location of the 3 target regions (*RiboPromoter*, *18S* and *28S*) that are amplified and analyzed by the MassARRAY EpiTYPER assay. Base positions are relative to the transcription starting site (+1) of rRNA primary transcript. For each amplicon the amplified strand is indicated, together with the sequence of the unconverted target region. The CpG sites whose methylation status can be assessed by the MassARRAY EpiTYPER assay are reported in bold. Abbreviations: 5’-ETS, 5’ external transcribed spacer; ITS1, Internal transcribed spacer 1; ITS2, Internal transcribed spacer 2; 3’-ETS, 3’ External transcribed spacer.

We used the MassARRAY EpiTYPER system to analyze the methylation status of three target regions (amplicons) in the rRNA gene (Figure 1): i) *RiboPromoter*, from position −186 to position +48 (respect to the transcription start site), including both the upstream and the core promoters of the gene; ii) *18S*, from position +2946 to position +3432, encompassing the 5’-sequence of the 18S region; iii) *28S*, from position +7297 to position +7579, encompassing the 5’-sequence of the 28S region. The three selected regions partially overlap with those previously analyzed in other studies [13,23].

The EpiTYPER assay returns quantitative methylation estimates of single CpGs or of small groups of adjacent CpGs (CpG units) depending on the sequence context. Using this method, we measured methylation levels of 8 CpG units (13 CpGs), 14 CpG units (26 CpGs) and 10 CpG units (15 CpGs) in *RiboPromoter*, *18S* and *28S* target regions respectively. In *RiboPromoter* amplicon, 7 CpGs were in the UCE region, while the remaining 6 were in the core promoter. The CpG units *RiboPromoter*_CpG_15.16 (UCE region) and *18S*_CpG_6.7 did not pass quality controls and were removed from further analysis.

### Assessment of rDNA methylation in normal and tumor tissues

**Figure 2:**
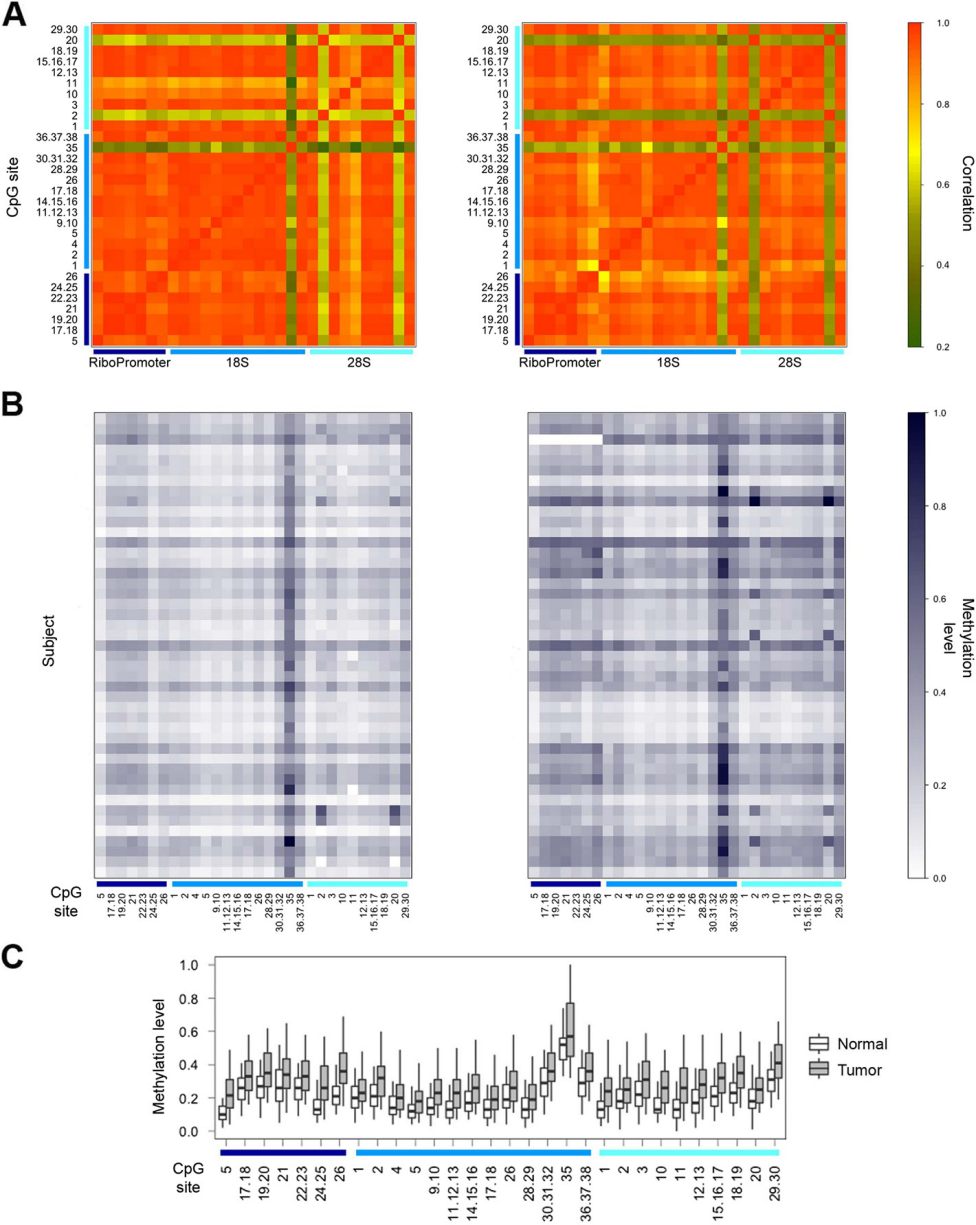
**DNA methylation of rDNA locus in pair-matched normal and tumor tissues. (A)** The correlation matrices of CpG sites analyzed in the 3 target regions (*RiboPromoter*, *18S* and *28S*) are reported for normal (left panel) and tumor (right panel) tissues. **(B)** The methylation levels of rDNA CpG sites are reported for 45 pair-matched normal (left panel) and tumor (right panel) tissues. **(C)** The boxplot compares, for each CpG site included in the analysis, the DNA methylation levels in 45 normal and tumor tissues.

We first considered the correlation between methylation values in the 45 samples for which both tumor and normal tissue were available (Figure 2A). As expected, most of the CpG units within the same target region showed high correlation. In addition, comparable high levels of correlation were detected also between CpG units in different amplicons, although they are several thousands of bases apart. Correlation levels were slightly but statistically significantly lower in tumor in respect to normal tissue (mean correlation values = 0.85 and 0.87 for tumor and normal tissue respectively, paired t-test p-value = 1.36 × 10^−10^).

CpG methylation values for each available normal-tumor tissue pair are graphically represented in Figure 2B. Considerable inter-individual variation was observed for each CpG unit both in normal and in tumor tissues. Despite this variability, we found highly significant hypermethylation in tumor in respect to matched normal breast tissue for all the analyzed CpGs (paired t-test, p-value ranging from 7.97 × 10^−12^ to 0.017 depending on the CpG unit; Figure 2C). Comparable significant hypermethylation of rDNA regions in tumors was evident also in the 22 samples with missing normal matched tissue (Additional file 1: Figure S1).

### Relationship between rDNA methylation and clinical and pathological parameters

**Figure 3:**
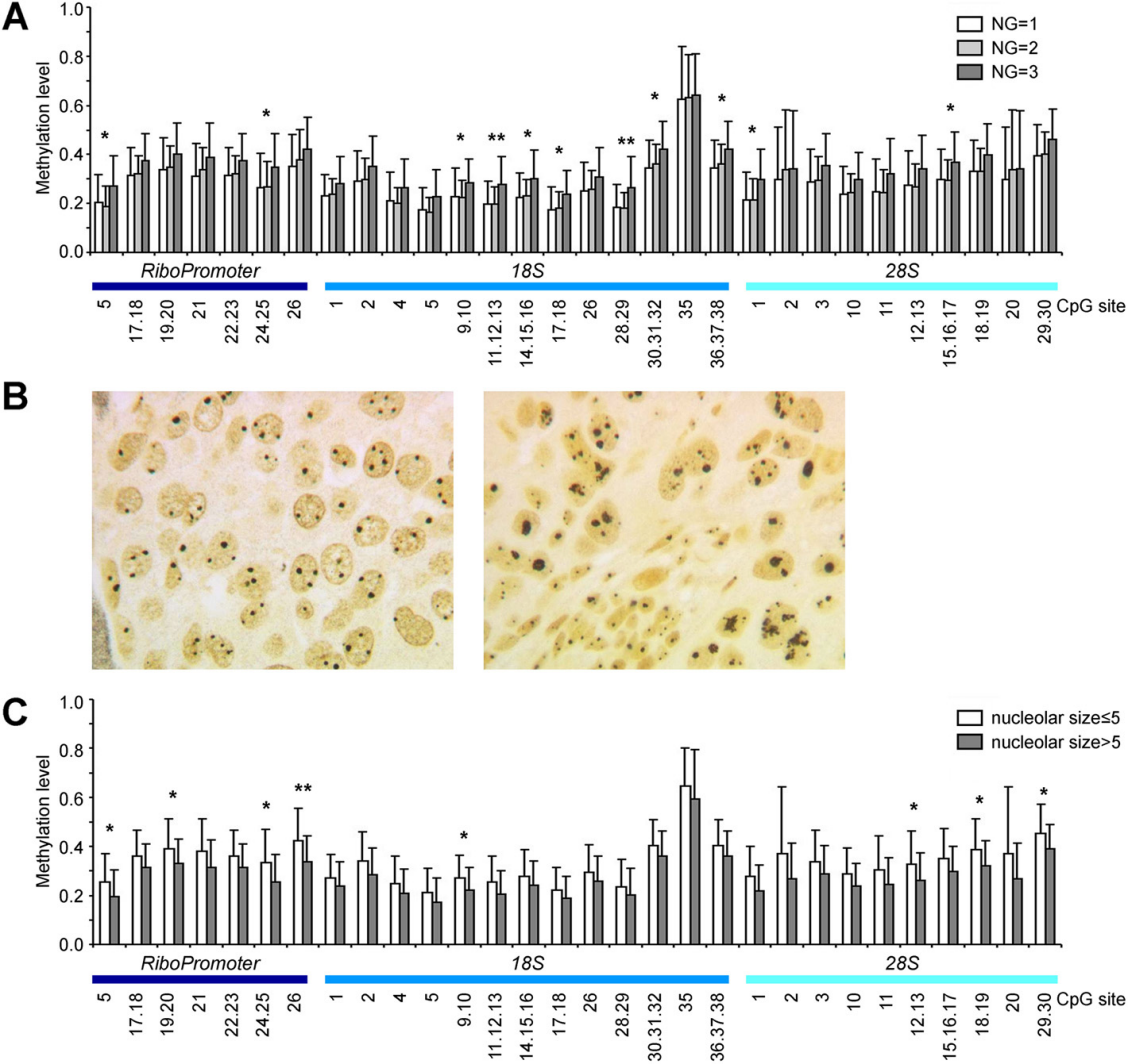
**Relationship between rDNA methylation and tumor parameters. (A)** Mean methylation levels of rDNA CpG sites in tumor samples divided for nuclear grade (NG). Standard deviation bars are reported. **(B)** Silver staining of two breast carcinomas. Note the higher quantity of silver stained nucleolar structures in left panel compared with those in right panel. **(C)** Mean methylation levels of rDNA CpG sites in tumor samples divided for nucleolar size values. Standard deviation bars are reported.

We recovered data on 66/68 tumors deeply characterized for clinical and pathological features (listed in Table 1) and, first of all, we investigated whether there was an association between this dataset and rDNA methylation profiles. Tumor samples were classified based on patient’s age, tumor histotype, size, grade (G), nuclear grade (NG), p53 status, ER and PR expression and proliferative index (Ki67) as indicated in Materials and Methods. We did not find significant differences in methylation status of rDNA between classes for investigated parameters, except for NG (Table 2). Indeed, a general trend towards rDNA hypermethylation was observed in NG = 3 samples respect to NG = 1 and NG = 2 samples (Figure 3A), with statistically significant differences (ANOVA analysis) for several CpG units within *RiboPromoter*; *18S* and *28S* (Table 2).

**Table 1:** Characteristics of the patients involved in the study

**Patient characteristics**	**N**
**Age** (years) (mean ± SD): 65.26 ± 12.68	
**Age at diagnosis:**	
≤ 50	16
> 50	52
**Histotype:**	
IDC	55
DC	2
ILC	8
IDLC	1
Undetermined	2
**Diameter** (mm) (mean ± SD): 18.90 ± 9.24	46
≤ 20	20
> 20	2
Undetermined	
**Tumor size (pT classification):**	
T1	46
T2	18
T3	2
Undetermined	2
**Tumor grade (G):**	
G1	18
G2	24
G3	24
Undetermined	2
**Nucleolar size (μm2):**	
≤ 5	23
> 5	41
Undetermined	4
**p53 expression (positivity):**	
≤ 10 (p53 wild type)	55
> 10 (mutated p53)	11
Undetermined	2
**ER expression:**	
Negative	11
Positive	55
Undetermined	2
**PR expression:**	
Negative	13
Positive	53
Undetermined	2
**Ki67 expression:**	
≥ 20	34
< 20	32
Undetermined	2

**Table 2:** ANOVA test of association between rDNA methylation and clinical and pathological parameters

**CpG unit**	**Age**	**Histotype**	**Grade**	**Nuclear grade**	**Diameter**	**pT**	**p53**	**ER**	**PR**	**Ki67**	**Nucleolar size**
Ribo_CpG_5	0.74	0.16	0.37	**0.02**	0.89	0.93	0.57	0.67	0.96	0.58	**0.04**
Ribo_CpG_17.18	0.70	0.12	0.28	0.08	0.70	0.74	0.79	0.62	0.91	1.00	0.09
Ribo_CpG_19.20	0.65	0.11	0.52	0.16	0.67	0.67	0.54	0.91	0.83	0.74	**0.04**
Ribo_CpG_21	0.72	0.09	0.33	0.13	0.61	0.59	0.55	0.92	0.73	0.77	0.05
Ribo_CpG_22.23	0.70	0.12	0.28	0.08	0.70	0.74	0.79	0.62	0.91	1.00	0.09
Ribo_CpG_24.25	0.92	0.20	0.38	**0.04**	0.55	0.75	0.57	0.95	0.80	0.88	**0.02**
Ribo_CpG_26	0.96	0.26	0.73	0.26	0.60	0.67	0.31	0.52	0.42	0.46	**0.01**
X18S_CpG_1	0.59	0.07	0.45	0.18	0.82	0.73	0.34	0.74	0.91	0.87	0.21
X18S_CpG_2	0.87	0.07	0.47	0.14	0.84	0.87	0.43	0.93	0.82	0.82	0.06
X18S_CpG_4	0.52	0.06	0.40	0.05	0.97	0.85	0.60	0.58	0.88	0.67	0.14
X18S_CpG_5	0.63	0.13	0.39	**0.04**	0.94	0.94	0.51	0.76	0.99	0.39	0.15
X18S_CpG_9.10	0.86	0.47	0.25	**0.04**	0.51	0.54	0.46	0.96	0.81	0.85	**0.05**
X18S_CpG_11.12.13	0.57	0.09	0.17	**0.01**	0.64	0.85	0.90	0.52	0.75	0.44	0.08
X18S_CpG_14.15.16	0.62	0.13	0.21	**0.02**	0.96	0.99	0.83	0.54	0.81	0.32	0.20
X18S_CpG_17.18	0.45	0.21	0.34	**0.03**	0.87	0.82	0.76	0.61	0.90	0.42	0.18
X18S_CpG_26	0.53	0.09	0.45	0.13	0.91	0.72	0.73	0.56	0.86	0.86	0.20
X18S_CpG_28.29	0.54	0.15	0.14	**0.01**	0.87	0.80	0.80	0.21	0.38	0.27	0.24
X18S_CpG_30.31.32	0.76	0.21	0.22	**0.05**	0.73	0.79	0.85	0.39	0.64	0.72	0.12
X18S_CpG_35	0.92	0.85	0.99	0.96	0.25	0.26	0.21	0.39	0.39	0.83	0.25
X18S_CpG_36.37.38	0.76	0.21	0.22	**0.05**	0.73	0.79	0.85	0.39	0.64	0.72	0.12
X28S_CpG_1	0.57	0.12	0.29	**0.02**	0.78	0.90	0.64	0.80	0.95	0.56	0.06
X28S_CpG_2	0.78	0.63	0.62	0.86	0.79	0.73	0.19	0.14	0.19	0.16	0.10
X28S_CpG_3	0.41	0.13	0.45	0.13	0.79	0.81	0.81	0.55	0.82	0.79	0.14
X28S_CpG_10	0.83	0.11	0.44	0.08	0.54	0.65	0.65	0.88	0.95	0.89	0.07
X28S_CpG_11	0.81	0.25	0.40	0.06	0.93	1.00	0.84	0.81	0.99	0.94	0.08
X28S_CpG_12.13	0.74	0.13	0.46	0.08	0.97	0.89	0.40	0.91	0.60	0.82	**0.05**
X28S_CpG_15.16.17	0.60	0.21	0.33	**0.03**	0.82	0.95	0.67	0.79	0.98	0.81	0.08
X28S_CpG_18.19	0.75	0.17	0.46	0.08	0.84	0.83	0.52	0.95	0.80	0.97	**0.03**
X28S_CpG_20	0.78	0.63	0.62	0.86	0.79	0.73	0.19	0.14	0.19	0.16	0.10
X28S_CpG_29.30	0.75	0.16	0.42	0.08	0.74	0.86	0.46	0.79	0.62	0.78	**0.04**

p-values less than 0.05 are reported in bold.

There is evidence that the quantitative distribution of the nucleolar organizer regions (NORs) after their selective staining with silver is closely related to the rates of rRNA transcription and of ribosome biogenesis, thus representing a morphological parameter of the rate of ribosome biogenesis [24-26]. We therefore focused on evaluating the relationship between the rate of ribosome biogenesis estimated by measuring the nucleolar size after selective silver staining of NORs and rDNA methylation. We successfully silver stained and measured 64/68 breast tissue specimens (Figure 3B). In order to compare the rDNA methylation levels respect to nucleolar size, we divided samples into two groups on the basis of this parameter: 41 samples showed a nucleolar area ≤ 5 μm^2^, whilst for 23 samples it was more than 5. We found that several CpG units within *RiboPromoter*, *18S* and *28S* rDNA regions were differently methylated between the two groups (Table 2, Figure 3C). For all CpG units we found that lower levels of rDNA methylation were associated to a higher rate of ribosome biogenesis (Figure 3C).

**Figure 4:**
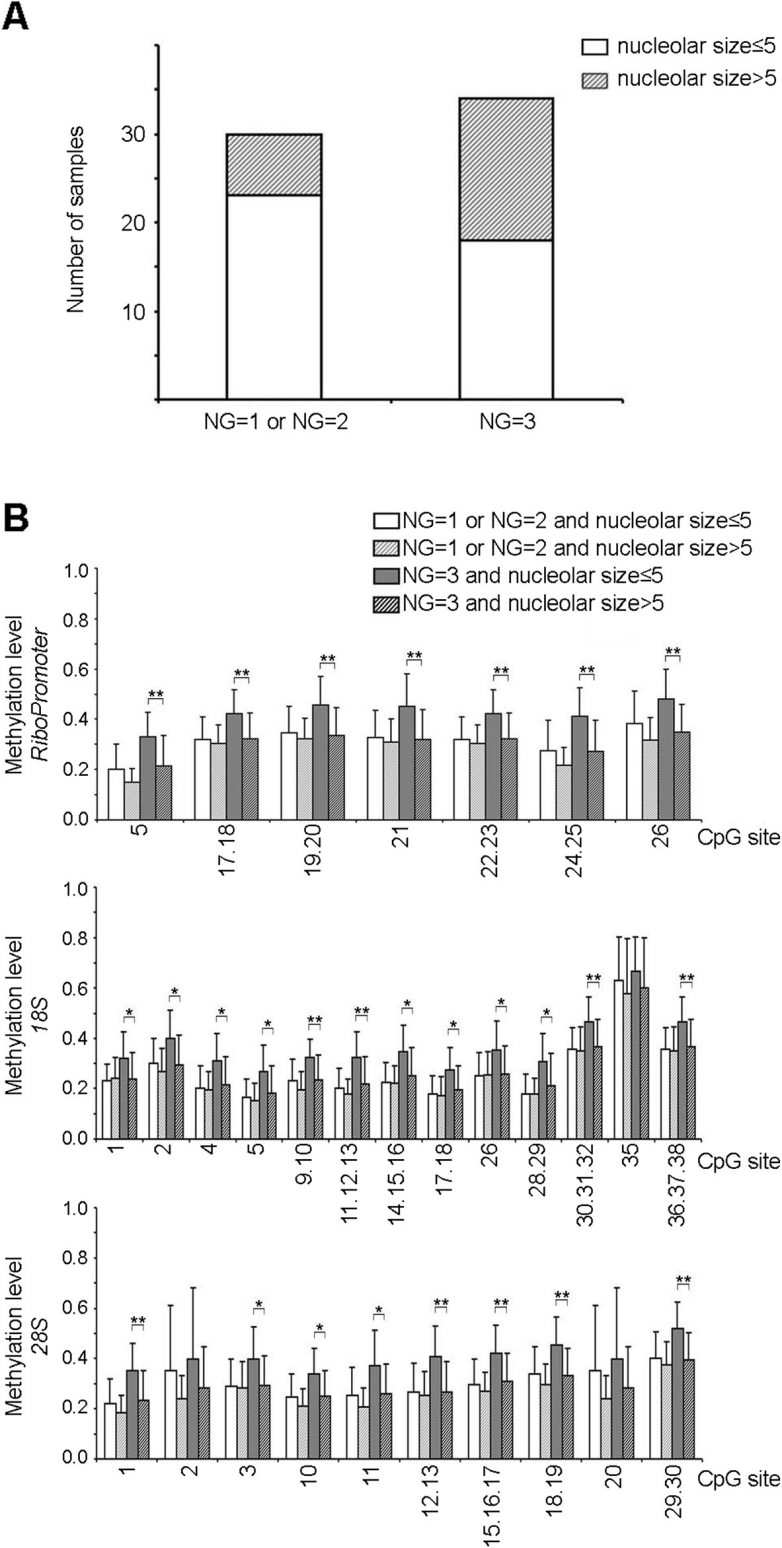
**Relationship between nuclear grade, nucleolar size and rDNA methylation. (A)** Classification of the analyzed breast carcinoma samples depending on NG and nucleolar size values. **(B)** Mean methylation levels of rDNA CpG sites in tumor samples divided in four classes depending on NG and nucleolar size values. Standard deviation bars are reported.

Considering that one of the parameters influencing nuclear grade classification is the presence of a prominent nucleolus, the results on nucleolar size appear in conflict with those on nuclear grade, being the average methylation of several sites of rDNA higher in NG3 tumors. To clarify this issue we evaluated the relationship between nuclear grade, nucleolar size and rDNA methylation. As previously observed [27,28], a nucleolar area > 5 occurred more frequently in samples with NG = 3 than in samples with NG = 1 o NG = 2 (Figure 4A; chi-squared test p-value *=* 0.048). Interestingly in NG = 1 and NG = 2 samples rDNA methylation levels were not significantly related to nucleolar size, while most of the CpG units resulted hypermethylated when a NG = 3 co-occurred with nucleolar size ≤ 5 μm^2^ (Figure 4B).

### Relationship between nucleolar size and rDNA methylation differences in tumor-normal tissue pairs

**Figure 5:**
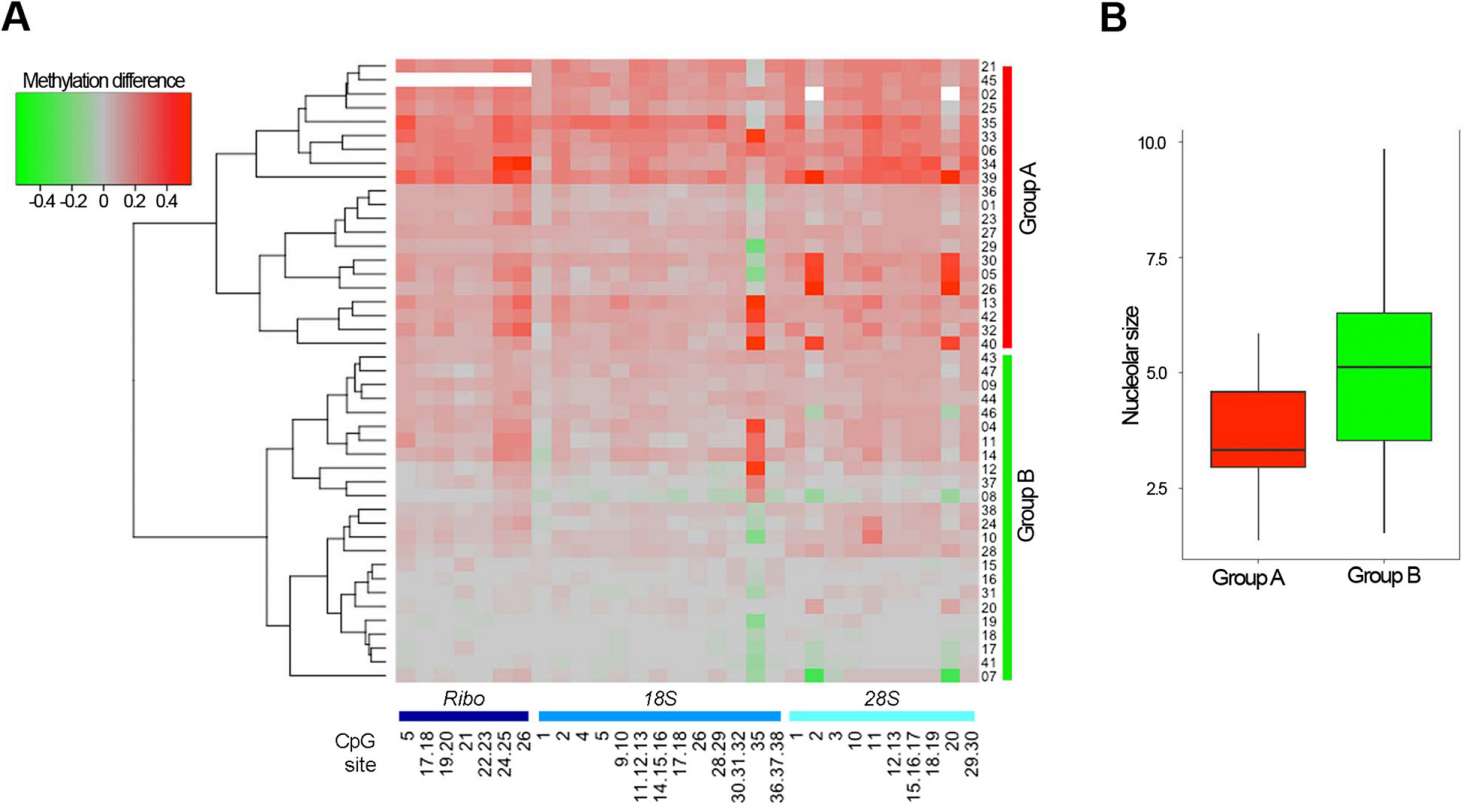
**Relationship between ribosome biogenesis and rDNA methylation differences in tumor-normal tissue pairs. (A)** For each normal-tumor tissue pair, DNA methylation differences were calculated and subjected to hierarchical clustering using complete linkage method and a euclidean distance measure. **(B)** The boxplot compares nucleolar size values between normal-tumor tissue pairs, subdivided in two groups on the basis of the results of hierarchical clustering.

Finally, we investigated whether irrespectively to the absolute value of rDNA methylation in tumor tissue, the extent of rDNA hypermethylation in tumor compared to normal matched tissue could be related to nucleolar size. To this purpose, for each CpG unit we calculated DNA methylation difference between tumor and matched normal tissue and performed hierarchical clustering analysis on these differences values (Figure 5A). Hierarchical clustering classified the 45 samples in 2 groups (indicated as A and B) ranging from low to marked rDNA hypermethylation of tumor tissue. Interestingly, group B comprised samples with very limited or absent rDNA hypermethylation in tumor tissue, indicating that increased rDNA methylation is not a feature shared by all breast carcinomas. Subsequently, we investigated if nucleolar areas were different in the 2 groups resulting from hierarchical clustering. ANOVA analysis showed that nucleolar size was significantly higher in group B samples (smaller DNA methylation difference in tumor-normal tissue pair, *i.e.*, lower hypermethylation in tumor samples) respect to group A samples (higher DNA methylation difference in tumor-normal tissue pair, *i.e.* stronger hypermethylation in tumor samples) (p-value = 0.006; Figure 5B). Similar results were achieved when only NG = 3 samples were considered (Additional file 2: Figure S2). No statistically significant differences were observed between group A and group B when the other clinical and pathological parameters were considered.

## Discussion

DNA methylation is a key regulator of gene expression and of genome architecture, and defects in its regulation often occur in several human diseases, including cancer. As many other types of tumors, up to 50% of cases of breast cancer show hypomethylation of repetitive DNA sequences and transposable elements, which substantially contributes to genomic instability [29]. Moreover, genome-wide studies on tumor tissues and breast cancer cell lines have reported aberrant hypermethylation of the CpG islands of several genes, including tumor suppressors [30-33].

In this study, we specifically analyzed the methylation of rDNA genes in breast cancer tissues. Indeed, altered regulation of ribosome biogenesis is a common feature of many cancers, and it has been deeply investigated in breast tumors [34]. In proliferating cancer cells, the rapidity of cell proliferation is strictly dependent on ribosome production [24,25,35]. This is one of the major factors contributing to the growth rate of a tumor mass inside the host, which is one of the most important prognostic factors in oncology. In human carcinomas the association of nucleolar hypertrophy with bad prognoses is noteworthy and there is an increasing amount of data that suggests an active role of the nucleolus in tumorigenesis [3,36]. In line with this, ribosome synthesis has been also identified as a promising target for antineoplastic therapy [37-44].

The methylation status of rDNA promoter, which is CpG-rich in human [45-47], was investigated in breast cancer tissues respect to matched normal tissues. The methylation of two CpG-rich regions located at the 5’ of 18S and 28S sequences was considered too. Our data indicate an increased rDNA methylation in tumors compared to normal tissues. Although this finding is unexpected, as neoplastic transformation should sustain ribosome biogenesis and therefore rDNA hypomethylation, similar results have been previously described. Yan and colleagues showed increased rDNA methylation levels in breast cancer biopsies compared to normal control tissue and found that rDNA hypermethylation was associated with the oestrogen receptor negative and with moderately or poorly differentiated tumors [19]. The technical approach employed in this work provided information about overall methylation status of rDNA, regardless to the epigenetic regulation of specific CpG sites [19]. Our results confirmed rDNA hypermethylation in breast tumors using the MassARRAY EpiTYPER assay, a technique that allows to assess methylation levels with single base resolution and that is more sensitive and quantitative compared to Southern blot and to clonal-sequencing of bisulfite-treated DNA. This approach allowed us to deeply characterize DNA methylation profile of rDNA. As expected, in normal tissues the CpG sites in the promoter showed a strong correlation in their methylation levels. Moreover, also the methylation status of the CpG sites within the gene body (18S and 28S regions) resulted highly correlated, suggesting a tight control over the entire region in normal conditions. Correlation levels were slightly but significantly lower in tumor samples respect to normal controls, indicating that a loss in the epigenetic control, which is a common characteristic of cancer, occurs also in rDNA region.

Surprisingly, rDNA methylation of normal breast tissues showed substantial inter-individual variation, ranging from 20% to 40% depending on the CpG site. The biological basis of this strong variability is not clear, although it should be considered that ribosomal biogenesis, and potentially also rDNA methylation, is strongly affected by environmental factors, such as the intracellular energy status [48].

Yan and coworkers showed that higher rDNA methylation levels in tumor breast tissues were correlated with ER-negativity and suggested that they could be predictive of the tumor propensity to hypermethylate ER promoter. We did not find any significant association between rDNA methylation and ER status, but it should be considered that in our cohort ER negative cases were only a minor part of the samples (11/68 compared to 27/58 in the work by Yan [19]). On the contrary, significant association was found between methylation values of several sites of rDNA loci and NG and nucleolar size values. Although only some CpGs reached statistical significance, the entire locus showed the same trend in terms of DNA methylation variations, confirming a common regulation of the CpG sites within the region. The nucleolar size evaluation after its selective staining with silver is a well established method used in tumor pathology for tumor characterization, being nucleolar hypertrophy associated with bad prognosis. Together with nuclear polymorphism, the presence of prominent nucleoli is one of the parameters influencing NG classification. We observed that a subgroup of samples with NG = 3 but nucleolar size ≤ 5 μm^2^ showed higher rDNA methylation levels, suggesting that in breast tumors the methylation status of rDNA loci can affect the rate of ribosome biogenesis and somehow counteract other adverse pathological conditions. Accordingly, we identified a subgroup of patients in which the presence of large nucleoli was associated to limited or absent rDNA hypermethylation of tumor tissue respect to matched normal control. In these tumors the lack of rDNA hypermethylation could represent an important factor to cope with the need for a particularly intense biosynthetic activity. In addition, this observation confirms an epigenetic regulation of ribosomal biogenesis in breast cancer and indicates that the rate of rDNA hypermethylation can significantly differ between patients. Importantly, samples showing small tumor-normal tissues differences had higher nucleolar size, indicating that not only the rDNA methylation level, but also the extent of rDNA hypermethylation in respect to normal tissue could represent a marker of breast cancer progression and, in principle, could be explored as a potential prognostic marker for this tumor type.

rDNA hypermethylation was described in other women’s cancers, including ovarian cancer [49] and endometrial carcinoma [50]. The mechanisms and the dynamics that lead to rDNA hypermethylation in these tumors are not clear, also because tumor progression should in theory sustain higher levels or ribosome biogenesis, and therefore rDNA hypomethylation in respect to normal tissue. In all the cases, higher levels of rDNA methylation were associated to better prognosis and longer disease-free and overall survival, suggesting that rDNA methylation could have a role in the biological and clinical behavior of the tumors. One intriguing scenario is that rDNA hypermethylation may be a defense response against tumor progression, but further analyses are needed to explore this issue. With respect to previous studies, where the relationship between rDNA methylation and ribosomal biogenesis was not considered, we demonstrated for the first time that rDNA methylation is associated to nucleolar size in breast cancer. Future studies should assess if rDNA methylation affects the rate of rRNA transcription and therefore the proliferative potential of tumor cells.

## Conclusions

In conclusion, in this study we showed that i) the methylation status of the CpG sites within the rDNA promoter and the 5’ of 18S and 28S sequences is tightly co-regulated in normal breast tissue, while in tumor tissue it is slightly but significantly lower; ii) rDNA methylation tends to be higher in breast cancer tissues respect to normal counterpart; iii) rDNA methylation levels are associated to NG and nucleolar size values and iv) in a subgroup of patients larger nucleolar size is associated with limited rDNA hypermethylation in tumor respect to matched normal tissue.

## Abbreviations

rDNA: Ribosomal DNA; rRNA: Ribosomal RNA; TSS: Transcription starting site; UCE: Upstream control element; G: Tumor grade; NG: Nuclear grade; AgNORs: Silver-stained Nucleolar Organizer regions.

## Competing interests

The authors declare that they have no competing interests.

## Authors’ contributions

LM, PG, SS, CF, MGB and AP conceived the study and wrote the article. MGB and AP developed the methodology. MGB, AP, MP, DT, CG, CP carried out the experimentation, acquired the data and performed statistical analysis. All authors read and approved the final manuscript for publication.

## Pre-publication history

The pre-publication history for this paper can be accessed here:

http://www.biomedcentral.com/1471-2407/14/361/prepub

## Supplementary Material

Additional file 1 **Supplementary Figure 1.** DNA methylation of rDNA locus in normal and unrelated tumor tissues. The boxplot compares, for each CpG site included in the analysis, the DNA methylation levels in 45 normal tissues and 23 unrelated tumor samples. Click here for file

Additional file 2 **Supplementary Figure 2.** Relationship between ribosome biogenesis and rDNA methylation differences in tumor-normal tissue pairs having NG = 3. (A) Only breast carcinomas with NG = 3 were considered. For each normal-tumor tissue pair, DNA methylation differences were calculated and subjected to hierarchical clustering. (B) The boxplot compares nucleolar size values between normal-tumor tissue pairs, subdivided in two groups on the basis of the results of hierarchical clustering. Click here for file

## References

[B1] Kopp K Gasiorowski JZ Chen D Gilmore R Norton JT Wang C Leary DJ Chan EKL Dean DA Huang S Pol I transcription and pre-rRNA processing are coordinated in a transcription-dependent manner in mammalian cells Mol Biol Cell 2007 18 394 403 17108330 10.1091/mbc.E06-03-0249 PMC1783775

[B2] Lempiäinen H Shore D Growth control and ribosome biogenesis Curr Opin Cell Biol 2009 21 855 863 10.1016/j.ceb.2009.09.002 19796927

[B3] Montanaro L Treré D Derenzini M Nucleolus, ribosomes, and cancer Am J Pathol 2008 173 301 310 10.2353/ajpath.2008.070752 18583314 PMC2475768

[B4] Schlesinger S Selig S Bergman Y Cedar H Allelic inactivation of rDNA loci Genes Dev 2009 23 2437 2447 10.1101/gad.544509 19833769 PMC2764490

[B5] Santoro R Li J Grummt I The nucleolar remodeling complex NoRC mediates heterochromatin formation and silencing of ribosomal gene transcription Nat Genet 2002 32 393 396 10.1038/ng1010 12368916

[B6] Tan BC-M Yang C-C Hsieh C-L Chou Y-H Zhong C-Z Yung BY-M Liu H Epigeneitc silencing of ribosomal RNA genes by Mybbp1a J Biomed Sci 2012 19 57 10.1186/1423-0127-19-57 22686419 PMC3407492

[B7] Ghoshal K Majumder S Datta J Motiwala T Bai S Sharma SM Frankel W Jacob ST Role of human ribosomal RNA (rRNA) promoter methylation and of methyl-CpG-binding protein MBD2 in the suppression of rRNA gene expression J Biol Chem 2004 279 6783 6793 14610093 10.1074/jbc.M309393200 PMC2242730

[B8] McStay B Grummt I The epigenetics of rRNA genes: from molecular to chromosome biology Annu Rev Cell Dev Biol 2008 24 131 157 10.1146/annurev.cellbio.24.110707.175259 18616426

[B9] Majumder S Ghoshal K Datta J Smith DS Bai S Jacob ST Role of DNA methyltransferases in regulation of human ribosomal RNA gene transcription J Biol Chem 2006 281 22062 22072 10.1074/jbc.M601155200 16735507 PMC2243234

[B10] Brown SE Szyf M Dynamic epigenetic states of ribosomal RNA promoters during the cell cycle Cell Cycle 2008 7 382 390 10.4161/cc.7.3.5283 18235221

[B11] Pietrzak M Rempala G Nelson PT Zheng J-J Hetman M Epigenetic silencing of nucleolar rRNA genes in Alzheimer’s disease PLoS One 2011 6 e22585 10.1371/journal.pone.0022585 21799908 PMC3142181

[B12] McGowan PO Sasaki A Huang TCT Unterberger A Suderman M Ernst C Meaney MJ Turecki G Szyf M Promoter-wide hypermethylation of the ribosomal RNA gene promoter in the suicide brain PLoS One 2008 3 e2085 10.1371/journal.pone.0002085 18461137 PMC2330072

[B13] Javierre BM Fernandez AF Richter J Al-Shahrour F Martin-Subero JI Rodriguez-Ubreva J Berdasco M Fraga MF O’Hanlon TP Rider LG Jacinto FV Lopez-Longo FJ Dopazo J Forn M Peinado MA Carreño L Sawalha AH Harley JB Siebert R Esteller M Miller FW Ballestar E Changes in the pattern of DNA methylation associate with twin discordance in systemic lupus erythematosus Genome Res 2010 20 170 179 10.1101/gr.100289.109 20028698 PMC2813473

[B14] Oakes CC Smiraglia DJ Plass C Trasler JM Robaire B Aging results in hypermethylation of ribosomal DNA in sperm and liver of male rats Proc Natl Acad Sci U S A 2003 100 1775 1780 10.1073/pnas.0437971100 12574505 PMC149909

[B15] Machwe A Orren DK Bohr VA Accelerated methylation of ribosomal RNA genes during the cellular senescence of Werner syndrome fibroblasts FASEB J 2000 14 1715 1724 10.1096/fj.99-0926com 10973920

[B16] Thomas G An encore for ribosome biogenesis in the control of cell proliferation Nat Cell Biol 2000 2 E71 E72 10.1038/35010581 10806485

[B17] Schmidt EV The role of c-myc in cellular growth control Oncogene 1999 18 2988 2996 10.1038/sj.onc.1202751 10378694

[B18] Uemura M Zheng Q Koh CM Nelson WG Yegnasubramanian S De Marzo AM Overexpression of ribosomal RNA in prostate cancer is common but not linked to rDNA promoter hypomethylation Oncogene 2012 31 1254 1263 10.1038/onc.2011.319 21822302 PMC3298623

[B19] Yan PS Rodriguez FJ Laux DE Perry MR Standiford SB Huang TH Hypermethylation of ribosomal DNA in human breast carcinoma Br J Cancer 2000 82 514 517 10.1054/bjoc.1999.0955 10682657 PMC2363317

[B20] Beahrs OH Pretreatment staging of cancer Cancer 1989 64 275 278 discussion 282–284 10.1002/1097-0142(19890701)64:1+<275::AID-CNCR2820641320>3.0.CO;2-J 2720615

[B21] Garagnani P Bacalini MG Pirazzini C Gori D Giuliani C Mari D Di Blasio AM Gentilini D Vitale G Collino S Rezzi S Castellani G Capri M Salvioli S Franceschi C Methylation of ELOVL2 gene as a new epigenetic marker of age Aging Cell 2012 11 1132 1134 10.1111/acel.12005 23061750

[B22] Trerè D AgNOR staining and quantification Micron 2000 31 127 131 10.1016/S0968-4328(99)00069-4 10588058

[B23] Espada J Ballestar E Santoro R Fraga MF Villar-Garea A Németh A Lopez-Serra L Ropero S Aranda A Orozco H Moreno V Juarranz A Stockert JC Längst G Grummt I Bickmore W Esteller M Epigenetic disruption of ribosomal RNA genes and nucleolar architecture in DNA methyltransferase 1 (Dnmt1) deficient cells Nucleic Acids Res 2007 35 2191 2198 10.1093/nar/gkm118 17355984 PMC1874631

[B24] Derenzini M Trerè D Pession A Montanaro L Sirri V Ochs RL Nucleolar function and size in cancer cells Am J Pathol 1998 152 1291 1297 9588897 PMC1858570

[B25] Derenzini M Trerè D Pession A Govoni M Sirri V Chieco P Nucleolar size indicates the rapidity of cell proliferation in cancer tissues J Pathol 2000 191 181 186 10.1002/(SICI)1096-9896(200006)191:2<181::AID-PATH607>3.0.CO;2-V 10861579

[B26] Hernandez-Verdun D The nucleolus: a model for the organization of nuclear functions Histochem Cell Biol 2006 126 135 148 10.1007/s00418-006-0212-3 16835752

[B27] Derenzini M Ceccarelli C Santini D Taffurelli M Treré D The prognostic value of the AgNOR parameter in human breast cancer depends on the pRb and p53 status J Clin Pathol 2004 57 755 761 10.1136/jcp.2003.015917 15220371 PMC1770366

[B28] Derenzini M Montanaro L Treré D What the nucleolus says to a tumour pathologist Histopathology 2009 54 753 762 10.1111/j.1365-2559.2008.03168.x 19178588

[B29] Soares J Pinto AE Cunha CV André S Barão I Sousa JM Cravo M Global DNA hypomethylation in breast carcinoma: correlation with prognostic factors and tumor progression Cancer 1999 85 112 118 10.1002/(SICI)1097-0142(19990101)85:1<112::AID-CNCR16>3.0.CO;2-T 9921982

[B30] Morita S Takahashi R-U Yamashita R Toyoda A Horii T Kimura M Fujiyama A Nakai K Tajima S Matoba R Ochiya T Hatada I Genome-wide analysis of DNA methylation and expression of micrornas in breast cancer cells Int J Mol Sci 2012 13 8259 8272 10.3390/ijms13078259 22942701 PMC3430232

[B31] Kikuyama M Takeshima H Kinoshita T Okochi-Takada E Wakabayashi M Akashi-Tanaka S Ogawa T Seto Y Ushijima T Development of a novel approach, the epigenome-based outlier approach, to identify tumor-suppressor genes silenced by aberrant DNA methylation Cancer Lett 2012 322 204 212 10.1016/j.canlet.2012.03.016 22433712

[B32] Rao X Evans J Chae H Pilrose J Kim S Yan P Huang R-L Lai H-C Lin H Liu Y Miller D Rhee J-K Huang Y-W Gu F Gray JW Huang T-M Nephew KP CpG island shore methylation regulates caveolin-1 expression in breast cancer Oncogene 2013 32 4519 4528 10.1038/onc.2012.474 23128390 PMC3787796

[B33] Faryna M Konermann C Aulmann S Bermejo JL Brugger M Diederichs S Rom J Weichenhan D Claus R Rehli M Schirmacher P Sinn H-P Plass C Gerhauser C Genome-wide methylation screen in low-grade breast cancer identifies novel epigenetically altered genes as potential biomarkers for tumor diagnosis FASEB J 2012 26 4937 4950 10.1096/fj.12-209502 22930747

[B34] Belin S Beghin A Solano-Gonzàlez E Bezin L Brunet-Manquat S Textoris J Prats A-C Mertani HC Dumontet C Diaz J-J Dysregulation of ribosome biogenesis and translational capacity is associated with tumor progression of human breast cancer cells PLoS One 2009 4 e7147 10.1371/journal.pone.0007147 19779612 PMC2744998

[B35] Derenzini M Montanaro L Chillà A Tosti E Vici M Barbieri S Govoni M Mazzini G Treré D Key role of the achievement of an appropriate ribosomal RNA complement for G1-S phase transition in H4-II-E-C3 rat hepatoma cells J Cell Physiol 2005 202 483 491 10.1002/jcp.20144 15389582

[B36] Montanaro L Treré D Derenzini M Changes in ribosome biogenesis may induce cancer by down-regulating the cell tumor suppressor potential Biochim Biophys Acta 1825 2012 101 110 10.1016/j.bbcan.2011.10.006 22079382

[B37] Bywater MJ Poortinga G Sanij E Hein N Peck A Cullinane C Wall M Cluse L Drygin D Anderes K Huser N Proffitt C Bliesath J Haddach M Schwaebe MK Ryckman DM Rice WG Schmitt C Lowe SW Johnstone RW Pearson RB McArthur GA Hannan RD Inhibition of RNA polymerase I as a therapeutic strategy to promote cancer-specific activation of p53 Cancer Cell 2012 22 51 65 10.1016/j.ccr.2012.05.019 22789538 PMC3749732

[B38] Drygin D Lin A Bliesath J Ho CB O’Brien SE Proffitt C Omori M Haddach M Schwaebe MK Siddiqui-Jain A Streiner N Quin JE Sanij E Bywater MJ Hannan RD Ryckman D Anderes K Rice WG Targeting RNA polymerase I with an oral small molecule CX-5461 inhibits ribosomal RNA synthesis and solid tumor growth Cancer Res 2011 71 1418 1430 10.1158/0008-5472.CAN-10-1728 21159662

[B39] Ruggero D Revisiting the nucleolus: from marker to dynamic integrator of cancer signaling Sci Signal 2012 5 pe38 22969157 10.1126/scisignal.2003477 PMC4390046

[B40] Drygin D Rice WG Grummt I The RNA polymerase I transcription machinery: an emerging target for the treatment of cancer Annu Rev Pharmacol Toxicol 2010 50 131 156 10.1146/annurev.pharmtox.010909.105844 20055700

[B41] Hein N Hannan KM George AJ Sanij E Hannan RD The nucleolus: an emerging target for cancer therapy Trends Mol Med 2013 19 643 654 10.1016/j.molmed.2013.07.005 23953479

[B42] Bywater MJ Pearson RB McArthur GA Hannan RD Dysregulation of the basal RNA polymerase transcription apparatus in cancer Nat Rev Cancer 2013 13 299 314 10.1038/nrc3496 23612459

[B43] Drygin D O’Brien SE Hannan RD McArthur GA Von Hoff DD Targeting the nucleolus for cancer-specific activation of p53 Drug Discov Today 2014 19 259 265 10.1016/j.drudis.2013.08.012 23993916

[B44] Montanaro L Treré D Derenzini M The emerging role of RNA polymerase I transcription machinery in human malignancy: a clinical perspective Onco Targets Ther 2013 6 909 916 23888116 10.2147/OTT.S36627 PMC3722134

[B45] Worton RG Sutherland J Sylvester JE Willard HF Bodrug S Dubé I Duff C Kean V Ray PN Schmickel RD Human ribosomal RNA genes: orientation of the tandem array and conservation of the 5’ end Science 1988 239 64 68 10.1126/science.3336775 3336775

[B46] Dante R Percy ME Baldini A Markovic VD Miller DA Rocchi M Niveleau A Miller OJ Methylation of the 5’ flanking sequences of the ribosomal DNA in human cell lines and in a human-hamster hybrid cell line J Cell Biochem 1992 50 357 362 10.1002/jcb.240500404 1281820

[B47] Gonzalez IL Wu S Li WM Kuo BA Sylvester JE Human ribosomal RNA intergenic spacer sequence Nucleic Acids Res 1992 20 5846 10.1093/nar/20.21.5846 1454549 PMC334433

[B48] Murayama A Ohmori K Fujimura A Minami H Yasuzawa-Tanaka K Kuroda T Oie S Daitoku H Okuwaki M Nagata K Fukamizu A Kimura K Shimizu T Yanagisawa J Epigenetic control of rDNA loci in response to intracellular energy status Cell 2008 133 627 639 10.1016/j.cell.2008.03.030 18485871

[B49] Chan MWY Wei SH Wen P Wang Z Matei DE Liu JC Liyanarachchi S Brown R Nephew KP Yan PS Huang TH-M Hypermethylation of 18S and 28S ribosomal DNAs predicts progression-free survival in patients with ovarian cancer Clin Cancer Res 2005 11 7376 7383 10.1158/1078-0432.CCR-05-1100 16243810

[B50] Powell MA Mutch DG Rader JS Herzog TJ Huang TH-M Goodfellow PJ Ribosomal DNA methylation in patients with endometrial carcinoma: an independent prognostic marker Cancer 2002 94 2941 2952 10.1002/cncr.10559 12115383

