# Supplementary figures and images for: The nucleolar size is associated to the methylation status of ribosomal DNA in breast carcinomas
Source: BMC Cancer. 2014 May 22;14:361. doi: 10.1186/1471-2407-14-361 (PMC4062283; doi:10.1186/1471-2407-14-361)

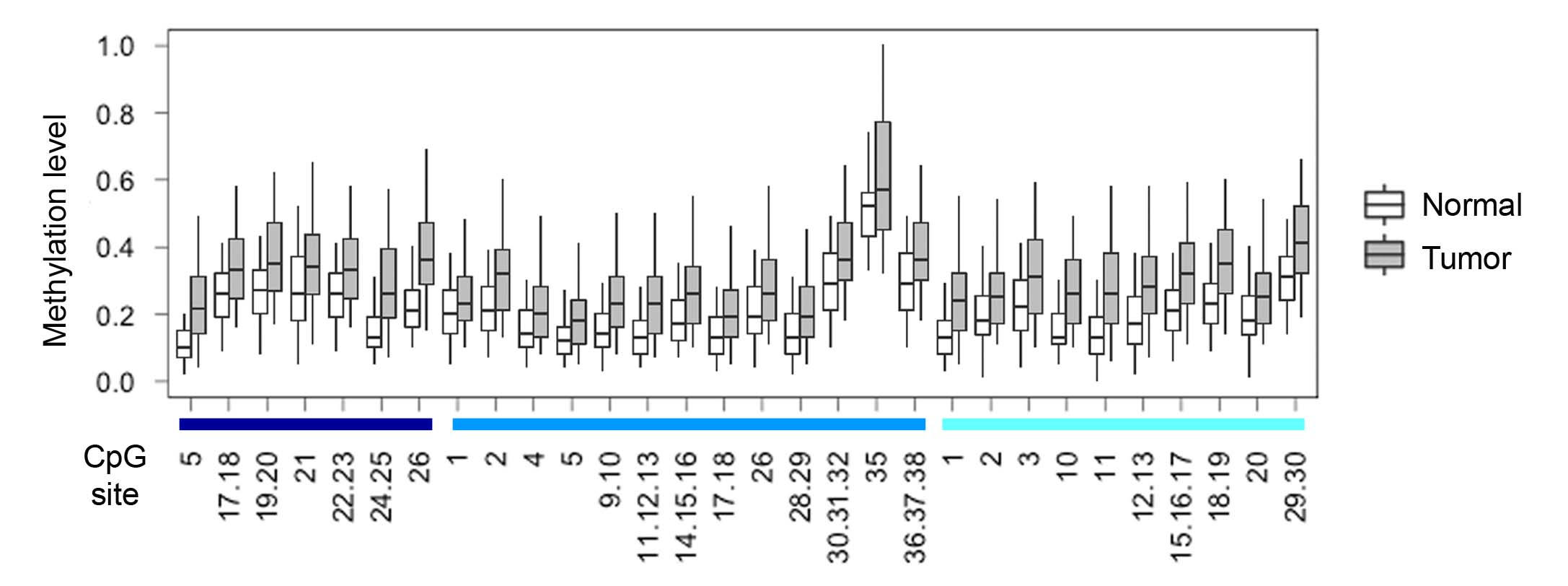

Supplement: Additional file 1 — Supplementary Figure 1. DNA methylation of rDNA locus in normal and unrelated tumor tissues. The boxplot compares, for each CpG site included in the analysis, the DNA methylation levels in 45 normal tissues and 23 unrelated tumor samples. [file 1471-2407-14-361-S1.jpeg]

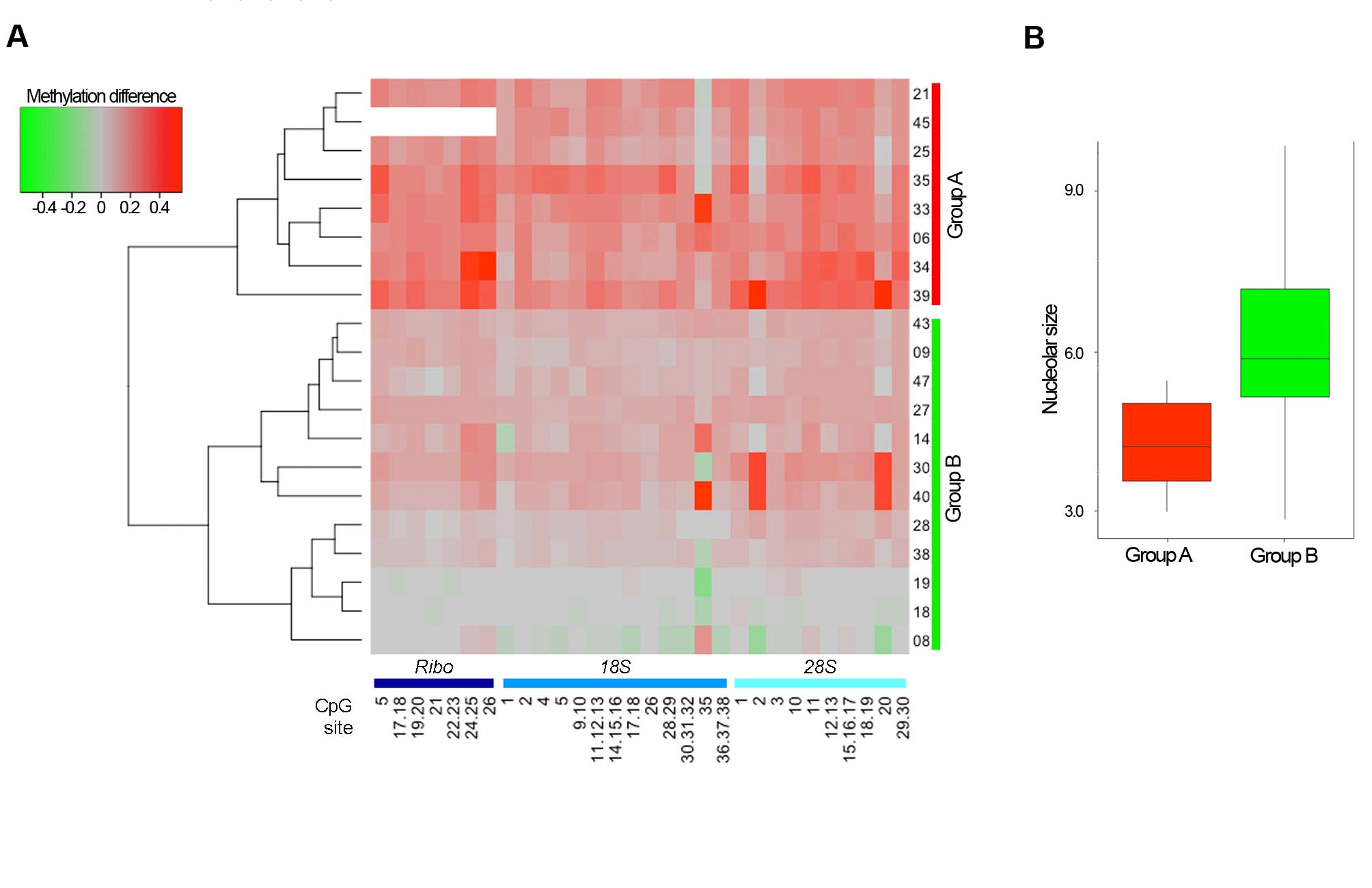

Supplement: Additional file 2 — Supplementary Figure 2. Relationship between ribosome biogenesis and rDNA methylation differences in tumor-normal tissue pairs having NG = 3. (A) Only breast carcinomas with NG = 3 were considered. For each normal-tumor tissue pair, DNA methylation differences were calculated and subjected to hierarchical clustering. (B) The boxplot compares nucleolar size values between normal-tumor tissue pairs, subdivided in two groups on the basis of the results of hierarchical clustering. [file 1471-2407-14-361-S2.jpeg]
